# Obesity and carotid atherosclerosis in African black and Caucasian women with established rheumatoid arthritis: a cross-sectional study

**DOI:** 10.1186/ar3784

**Published:** 2012-03-19

**Authors:** Ahmed Solomon, Gavin R Norton, Angela J Woodiwiss, Patrick H Dessein

**Affiliations:** 1 Department of Rheumatology, Charlotte Maxeke Johannesburg Academic Hospital, Faculty of Health Sciences, University of the Witwatersrand, 7 York Road, Parktown, Johannesburg 2193, South Africa; 2 Cardiovascular Pathophysiology and Genomics Research Unit, School of Physiology, Faculty of Health Sciences, University of the Witwatersrand, 7 York Road, Parktown, Johannesburg 2193, South Africa

## Abstract

**Introduction:**

Reported findings on the relationship between adiposity and atherosclerotic cardiovascular disease (ACVD) risk in rheumatoid arthritis (RA) are contradictory and originate in developed populations. Approximately 80% of ACVD now occurs in developing countries. We aimed to ascertain the associations of clinical obesity measures with metabolic cardiovascular risk and atherosclerosis in African women with RA from a developing black and developed Caucasian population.

**Methods:**

The associations of body mass index (BMI) as an indicator of overall adiposity and waist circumference and waist-to-height and waist-to-hip ratios as abdominal obesity indices with metabolic risk factors and high resolution B-mode ultrasound-determined carotid artery atherosclerosis were assessed in multivariate regression models in 203 African women with established RA; 108 were black and 95 Caucasian.

**Results:**

BMI and waist-to-height ratio were higher in African black compared to Caucasian women (29.9 (6.6) versus 25.3 (4.9) kg/m^2^, *P *= 0.002 and 0.59 (0.09) versus 0.53 (0.08), *P *= 0.01, respectively). Interactions between population origin and anthropometric measures were not related to metabolic risk factors but were associated with atherosclerosis, independent of confounders and individual terms. In all patients, BMI was related to systolic and diastolic blood pressure but not with serum lipid concentrations whereas abdominal obesity indices were associated with serum lipid concentrations but not with blood pressure values; obesity measures that were associated with plasma glucose concentrations comprised BMI, waist circumference and waist-to-height ratio (*P *< 0.05 in multiple confounder adjusted analysis). In African Caucasian women, BMI was associated with common carotid artery intima-media thickness (standardized β (95% confidence interval (CI)) = 0.21 (0.03 to 0.38)) and waist-to-hip ratio with plaque (odds ratio (OR) (95% CI) = 1.83 (1.03 to 3.25) for one standard deviation (SD) increase). These relationships were independent of multiple non-metabolic risk factors and explained by metabolic risk factors. In African black women with RA, none of the obesity measures was related to atherosclerosis.

**Conclusions:**

Obesity in women with RA from developing groups of black African descent does not as yet translate into atheroma. In Caucasian women with RA that belong to developed populations, BMI and waist-to-hip ratio should be considered in ACVD risk assessment.

## Introduction

Rheumatoid arthritis (RA) is a chronic inflammatory and potentially destructive joint disease that enhances the risk for atherosclerotic cardiovascular disease (ACVD) event rates two fold [1]. ACVD death rates are increased by 50% and responsible for most of the excess mortality in RA [2]. Traditional and nontraditional cardiovascular risk factors as well as genetic polymorphisms were each reported to be associated with cardiovascular disease in RA [3-12].

The role of excess adiposity in ACVD and cardiovascular risk assessment among persons with RA has become a controversial issue. Whereas adiposity as assessed by the body mass index (BMI) and waist circumference was shown to be independently associated with enhanced metabolic cardiovascular risk [13,14], a paradoxical inverse relationship between the BMI and ACVD and overall mortality were reported in RA [15,16]. The increased ACVD risk associated with a low BMI in patients with RA could be explained by chronic inflammation and inactivity mediated reduced lean mass and particularly muscle mass in the presence of increased body fat accumulation, a condition known as rheumatoid cachexia [15-17]. Alternatively, Stavropoulos-Kalinoglou and colleagues recently proposed that a paradoxical epidemiological association between survival outcomes and cardiovascular risk factors such as obesity as well as analytical deficiencies in some of the previous studies may account for the reported seemingly contradictory observations on the potential impact of adiposity on cardiovascular risk in RA [18]. Importantly in the present context, BMI constitutes the only obesity measure in RA studies on cardiovascular morbidity and mortality [18]. However, BMI does not discriminate between body fat percentage and lean mass [19] and predicts cardiovascular disease less effectively than measures of central obesity [20-22] in non-RA subjects. To the best of our knowledge, the relative role of different clinical obesity measures in the assessment of ACVD risk in RA has not been reported.

Reported data on atherogenesis in the general population and diseases such as RA derive almost exclusively from developed populations [23,24]. Nevertheless, approximately 80% of ACVD now arises in low income or developing countries. In South Africa, a sub-Saharan country, a minority of citizens consist of an overall affluent, developed and mostly Caucasian population whereas the majority are socially and economically disadvantaged, at an earlier epidemiological health transition stage with consequent different cardiovascular risk factor profiles and disease presentation and mostly of black African ancestry [25-27]. A markedly high prevalence of obesity has been increasingly reported especially in black South African women [28,29]. Still, ACVD event rates remain distinctly low in black Africans [25-27,30]. These findings suggest that in populations that are at an earlier epidemiological transition stage, obesity may not as yet translate into atheroma. Whether this apparent lack of association between excess adiposity and ACVD is present in persons with RA from developing populations, is currently unknown. In the present study, we determined disparities in the relationships of obesity measures with atherosclerosis between women with RA from a developing black compared to a developed Caucasian population. In addition, we examined the potential role of different clinical obesity measures including BMI, waist circumference and waist-to-height and waist-to-hip ratios in assessing metabolic cardiovascular risk and ultrasonographically determined carotid artery atherosclerosis among women with RA.

## Materials and methods

### Study populations

We enrolled African black and Caucasian patients who met the American College of Rheumatology criteria for RA [31] at the Charlotte Maxeke Johannesburg Academic Hospital (public healthcare) and Milpark Hospital (private healthcare) in Johannesburg. None of the data have been reported previously. Four invited patients refused to enroll. Only 13 black African men with RA participated and hence to avoid confounding of the data analyses by gender differences between black and Caucasian patients with RA, the data analysis was performed in women only. Patients who had used disease modifying agents, that is, persons with established RA, were included and those known to be infected with HIV were excluded. The study was approved by the Ethics Committee for Research on Human Subjects (Medical) of the University of the Witwatersrand. Written informed consent was obtained from each patient.

### Assessments

Using methods previously reported by us [3,14], we assessed sociodemographic characteristics, lifestyle factors, systolic and diastolic blood pressure, serum lipid and glucose concentrations, RA characteristics, markers of systemic inflammation including the erythrocyte sedimentation rate (ESR) and C-reactive protein (CRP) concentrations and other potential cardiovascular risk factors comprising years of education, thyroid status and hormone replacement therapy. Exercise included hours spent in walking (for example, to reach public transportation). Data were missing in fewer than 5% for any of the recorded variables. Serum lipid and plasma glucose concentrations were determined on fasting blood samples using standard laboratory methods.

BAS (see acknowledgements) and AS performed the carotid artery ultrasound measurements in private and public healthcare patients, respectively. Both operators obtained images of at least 1 cm length of the distal common carotid arteries for measurement of the intima-media thickness of the far wall from an optimal angle of incidence defined as the longitudinal angle of approach where both branches of the internal and external carotid artery are visualized simultaneously [32] and with high resolution B-mode ultrasound (Image Point, Hewlett Packard, Andover, MA, USA and SonoCalc IMT, Sonosite Inc, Bothell, Wash, USA used by BAS and AS, respectively) employing linear array 7.5 MHz probes. The details of the methodology used by BAS were reported previously [3]. The equipment used by AS involves the application of a unique semi-automated border detection program that was previously found to provide highly reproducible results [32]. The intima-media thicknesses in the left and right common carotid artery were measured and the carotid intima-media thickness (cIMT) was defined as the mean of these. Carotid artery plaque was defined as a focal structure that encroaches into the arterial lumen of at least 0.5 mm or 50% of the surrounding intima-media thickness value, or demonstrates a thickness of > 1.5 mm as measured from the media-adventitia interface to the intima-lumen interface [33]. Both operators were blinded to the cardiovascular risk profiles of the patients. Repeat ultrasound examinations by both operators on 23 patients revealed Spearman correlations between repeat cIMT measurements of 0.983 and 0.956 for BAS and AS, respectively, and the correlation between measurements made by BAS and AS was 0.926. Both operators identified carotid artery bulb or/and internal carotid artery plaque in 11 of these 23 patients with full inter-observer agreement.

Height and weight were measured with participants wearing light clothing and no shoes and the BMI was calculated using these parameters. Waist circumference was measured at the umbilical level and hip at the level of the largest circumference. The adiposity measures included in our main data analysis comprised BMI as an indicator of overall obesity and waist circumference and waist-to-height and waist-to-hip ratios as abdominal obesity indices, respectively [20-22]. The relationships between waist-to-height ratio and incident ACVD and diabetes are reportedly most consistent across different population groups [22].

### Statistical analysis

Continuous variables are reported as mean (SD) and categorical variables as proportions or percentages. Non-normally distributed characteristics were logarithmically transformed prior to statistical analysis and for these variables geometric means (SD) are given.

Disparities in sociodemographic features between African black and Caucasian women were compared using the Student t-test and univariate logistic regression analysis as appropriate. Relationships of population grouping (PG) with baseline characteristics, cIMT and carotid artery plaque and anthropometric measures were investigated in multivariate logistic and linear regression models as appropriate and with consistent adjusting for age and healthcare center attendance as well as lipid lowering, antihypertensive and glucose lowering agents in models that included lipid, systolic and diastolic blood pressure and glucose variables, respectively.

Disparities in the relationships of obesity measures with metabolic risk factors and with atherosclerosis in African black compared to Caucasian women were identified by assessing the associations of interactions between PG and obesity measures with metabolic risk factors and cIMT and plaque, respectively, in confounder and individual term adjusted multivariate regression models; when significant interactions were present, stratified analysis was performed.

Finally, we determined whether associations of obesity measures with carotid atherosclerosis as identified in the above mentioned analyses were independent of multiple non-metabolic risk factors and explained by metabolic risk factors in additional multivariate regression models.

Statistical computations were made using the GB Stat™ program (Dynamic Microsystems, Inc, Silverspring, Maryland, USA).

## Results

### Baseline characteristics and atherosclerosis in African black compared to Caucasian women with RA

Baseline characteristics and carotid atherosclerosis in African black and Caucasian women with RA are shown in Table 1. African black women were on average 2.1 years younger (*P *= 0.2) than their Caucasian counterparts. Approximately 97% of African black women and 80% of Caucasian women attended the public and private healthcare center, respectively (*P *< 0.0001). As compared to African Caucasian women and in confounder adjusted analysis, black women with RA had a smaller pack year history of smoking, used alcohol less often, exercised less, had lower total and HDL cholesterol concentrations but similar LDL cholesterol, triglycerides and non-HDL cholesterol concentrations as well as cholesterol÷HDL cholesterol and triglycerides÷HDL cholesterol ratios, higher plasma glucose concentrations, more deformed joints, higher ESRs and lower educational levels. Ever prednisone use was similar in African black and Caucasian women in univariate (*P *= 0.94) and adjusted analysis (*P *= 0.08, see Table 1). The small proportion of current prednisone users reflects our previously reported increasing avoidance of this intervention [24].

**Table 1:** Baseline characteristics and atherosclerosis in Caucasian and black women with RA

Characteristic	Caucasian women (*n *= 95)	Black women (*n *= 108)	*P* ^a^
Sociodemographics			
Age, years	57.5 (11.7)	55.4 (10.0)	...
Public healthcare (%)	18.9	97.2	...
Lifestyle factors			
Pack year history smoking, n	2.4 (5.0)	0.1 (1.5)	0.003
Current smoking (%)	9.6	1.9	0.04
Never smoking (%)	90.7	58.5	0.01
Former smoking (%)	31.9	8.4	0.01
Alcohol use (%)	34.0	1.9	< 0.0001
Exercise, hours per week^b^, n	0.7 (2.1)	0.6 (1.9)	0.04
Blood pressure			
Systolic blood pressure, mmHg	128 (17)	140 (24)	0.5
Diastolic blood pressure, mmHg	78 (9)	85 (15)	0.1
Lipids			
Total cholesterol, mmol/l	5.1 (1.0)	4.7 (0.9)	0.02
LDL cholesterol, mmol/l	2.8 (0.9)	2.6 (0.8)	0.5
HDL cholesterol^b^, mmol/l	1.7 (1.3)	1.5 (1.3)	0.002
Cholesterol÷HDL cholesterol	3.1 (1.0)	3.3 (1.1)	0.1
Triglycerides^b^, mmol/l	1.1 (1.5)	1.1 (1.7)	0.8
Triglycerides÷HDL cholesterol^b^	0.6 (1.8)	0.7 (2.1)	0.1
Non-HDL cholesterol	3.3 (1.0)	3.2 (0.9)	0.5
Glucose^b^, mmol/l	4.7 (1.2)	5.2 (1.4)	0.02
Diabetes mellitus (%)	4.2	16.7	0.3
RA characteristics			
Disease duration, years	12.7 (9.1)	14.8 (9.4)	0.6
Rheumatoid factor positive (%)	73.4	75.9	0.8
DAS28	3.5 (1.5)	4.1 (1.3)	0.5
Deformed joints^b^, n	4 (4)	7 (3)	0.05
Prednisone use ever (%)	43.2	42.6	0.08
Current prednisone use (%)	4.2	1.9	0.07
Current DMARD, n	2.1 (0.9)	2.5 (1.0)	0.4
Current methotrexate use (%)	77.9	91.7	0.9
Current chloroquine use (%)	47.4	77.8	0.2
Systemic inflammation			
Erythrocyte sedimentation rate^b^, mm/hr	7 (3)	21 (3)	0.007
C-reactive protein^b^, mg/l	3.9 (3.6)	7.5 (3.1)	0.5
Cardiovascular drugs			
Antihypertensive agent use (%)	43.2	55.6	0.07
Oral glucose lowering agent use (%)	4.2	13.9	0.03
Insulin use (%)	1.1	2.0	0.6
Statin use (%)	36.8	18.5	0.03
Ezetimibe use (%)	2.1	0	...
Other			
Education, years	12.7 (2.7)	7.5 (4.1)	0.002
Hypothyroidism^c ^(%)	34.7	6.5	0.2
Hormone replacement therapy (%)	17.9	5.6	0.8
Atherosclerosis			
cIMT, mm	0.689 (0.117)	0.691 (0.099)	0.6
Plaque (%)	36.8	35.2	0.5

Results are expressed as mean (SD) or proportions/percentages. ^a^*P *for comparison between black and Caucasian women with RA after adjustment for age and healthcare as well as lipid lowering and antihypertensive agents in models that include lipid and blood pressure variables, respectively; ^b^non-normally distributed variable for which geometric mean (SD) is given; ^c ^includes subclinical and overt hypothyroidism and diagnosed when serum thyrotropin level was > 4.94 mU/L or when thyroid hormone replacement therapy was used. cIMT, common carotid artery intima-thickness; DAS28, Disease Activity Score in 28 joints; DMARD, disease modifying agents for rheumatic disease; n, number of; RA, rheumatoid arthritis.

### Anthropometric measures in African black compared to Caucasian women with RA

The anthropometric measures in African black and Caucasian women with RA are shown in Table 2. In age and healthcare center adjusted analysis, African black women had higher body weight, BMI and waist-to-height ratio compared to their Caucasian counterparts; by contrast, height, waist circumference, hip circumference and the waist-to-hip ratio were similar in both groups.

**Table 2:** Anthropometric measures in Caucasian and black women with RA

Anthropometric measures	Caucasian women (*n *= 95)	Black women	*P* ^a^ (*n *= 108)
Height, cm	163 (7)	159 (7)	0.08
Body weight, kg	67.2 (15.2)	75.8 (16.3)	0.03
BMI, kg/m^2^	25.3 (4.9)	29.9 (6.6)	0.002
Waist circumference, cm	87 (13)	94 (14)	0.06
Waist÷height	0.53 (0.08)	0.59 (0.09)	0.01
Hip circumference^b^	101 (1)	110 (1)	0.2
Waist÷hip	0.84 (0.08)	0.85 (1.12)	0.4

Results are expressed as mean (SD). ^a^*P*-value adjusted for age and healthcare center; ^b ^non-normally distributed variable for which geometric mean (SD) is given. BMI, body mass index; RA, rheumatoid arthritis.

### Relationships of obesity measures with metabolic risk factors in African black and Caucasian women with RA

The relationships between obesity measures and metabolic risk factors in all women with RA are shown in Table 3. Potentially confounding characteristics that were included as independent variables in the respective regression models comprised age, healthcare center, lifestyle factors (smoking variable was pack-year history of smoking), current disease activity (DAS28), cumulative disease activity (deformed joints), prednisone use, hypothyroidism and hormone replacement therapy as well as antihypertensive, lipid and glucose lowering agents upon entering of blood pressure, lipid and glucose parameters, respectively. Interactions between PG and obesity measures were consistently unrelated to metabolic risk factors and therefore no stratified analysis was performed. BMI was associated with blood pressure variables but not with serum lipid concentrations. By contrast, waist circumference and waist-to-height and waist-to-hip ratios were related to serum lipid concentrations but not to blood pressure values. Obesity measures that were associated with plasma glucose concentrations comprised BMI, waist circumference and waist-to-height ratio.

**Table 3:** Associations between obesity measures and metabolic risk factors in 203 African women with RA

Metabolic risk factor		Obesity measure		
	
	BMI	Waist	Waist÷height	Log waist÷hip
Systolic blood pressure	**0.23 (0.09 to 0.37)^a^**	0.11 (-0.01 to 0.25)	0.09 (-0.06 to 0.24)	-0.05 (-0.16 to 0.07)
Diastolic blood pressure	**0.29 (0.15 to 0.43)^b^**	0.13 (-0.13 to 0.28)	-0.05 (-0.18 to 0.26)	0.10 (-0.05 to 0.09)
Log triglycerides	0.11 (-0.06 to 0.25)	**0.27 (0.14 to 0.41)^b^**	**0.28 (0.13 to 0.43)^b^**	0.14 (0.00 to 0.29)
Log HDL cholesterol	-0.02 (-0.19 to 0.14)	**-0.22 (-0.33 to -0.08)^b^**	**-0.20 (-0.36 to -0.04)^a^**	**-0.21 (-0.35 to -0.07)^b^**
Log glucose	**0.16 (0.02 to 0.27)^a^**	**0.15 (0.01 to 0.30)^a^**	**0.18 (0.04 to 0.33)^a^**	0.02 (-0.10 to 0.13)

Results are expressed as standardized regression coefficients (95% confidence interval) in multivariable models in which age, healthcare center, lifestyle factors, current disease activity (DAS28), cumulative disease activity (log deformed joints), prednisone use, hypothyroidism and hormone replacement therapy as well as antihypertensive, lipid and glucose lowering agents upon entering of blood pressure, lipid and glucose parameters as independent variables, respectively, were controlled for. Significant associations are shown in bold. ^a^*P*-value < 0.05; ^b^*P*-value < 0.01. BMI, body mass index; HDL, high-density lipoprotein; Log, logarithmically transformed.

The selection of confounders modeled in the analysis shown in Table 3 was based on biological plausibility and not data driven. The associations of these characteristics with obesity measures in African black and Caucasian women are shown in Table 4. Age, smoking history, deformed joints and cardiovascular drugs were each associated with obesity measures in African black or/and Caucasian women. Additionally, there were several disparities in these relationships between African black and Caucasian participants. Age was associated with a high waist-to-hip ratio and smoking with a high BMI, waist circumference and waist-to-height ratio in African Caucasian but not in black women. By contrast, the number of deformed joints was associated with a low BMI, waist circumference and waist-to-height ratio in African black but not in Caucasian women.

**Table 4:** Associations between obesity measures and non-metabolic risk factors in African black and Caucasian women with RA

Non-metabolic risk factor	Obesity measure											
	
	BMI			Waist			Waist÷height			Log waist÷hip		
				
	Black	Caucasian	Interaction	Black	Caucasian	Interaction	Black	Caucasian	Interaction	Black	Caucasian	Interaction
			*P*-value			*P*-value			*P*-value			*P*-value
Age	-0.09	-0.10	0.9	-0.10	-0.01	0.4	-0.04	0.10	0.6	-0.09	**0.38^b^**	**0.004**
Public healthcare	-0.05	0.07	0.4	-0.08	-0.01	0.3	-0.04	0.13	0.4	-0.07	-0.04	0.5
L py history smoking	-0.16	**0.22^a^**	**0.03**	-0.14	**0.27^a^**	0.05	-0.15	**0.25^a^**	**0.048**	0.08	0.18	0.6
Alcohol use	0.07	0.05	0.08	0.06	0.07	**0.04**	0.03	0.06	0.08	-0.08	-0.02	0.5
Exercise	-0.00	-0.07	0.7	0.05	-0.12	0.2	0.06	-0.16	0.1	-0.03	-0.04	0.9
DAS28	-0.06	0.19	0.2	0.03	0.11	0.5	0.03	0.16	0.4	-0.04	0.07	0.4
L deformed joints	**-0.25^a^**	-0.15	0.2	**-0.32^b ^**	-0.06	**0.01**	**-0.28^b^**	-0.08	**0.036**	-0.11	-0.10	0.2
Prednisone use	-0.10	-0.07	0.6	-0.15	0.02	0.2	-0.16	-0.05	0.5	-0.09	0.05	0.4
Hypothyroidism	-0.11	0.16	0.1	0.04	0.08	0.9	-0.04	0.11	0.4	0.05	0.03	0.7
HRT	0.04	-0.06	0.6	-0.02	-0.07	0.8	-0.04	-0.07	0.9	-0.09	-0.04	0.5
Anti-HTA use	0.15	**0.35^b^**	0.5	**0.23^a^**	**0.30^†^**	0.5	0.19	**0.29^b^**	0.6	0.00	**0.23^a^**	**0.039**
Statin use	0.10	**0.30^b^**	0.4	0.10	**0.29^b^**	0.8	0.05	**0.28^b^**	0.2	0.06	0.06	0.8
Ezetimibe use	...	0.17	...	...	0.08	...	...	0.11	...	...	0.02	...
OHA use	**0.27^b^**	**0.25^a^**	0.5	**0.31^b^**	**0.25^a^**	0.5	**0.29^b^**	**0.26^a^**	0.7	0.00	0.14	0.3
Insulin therapy	**0.25^b^**	-0.05	0.06	**0.26^a^**	-0.03	0.07	**0.30^b^**	-0.05	**0.042**	0.09	0.03	0.6

Results are expressed as standardized regression coefficients in univariate analysis for the relationships between obesity measures and age and public healthcare and in age and public healthcare adjusted analysis for the associations between obesity measures and the other non-metabolic risk factor measures. Significant associations are shown in bold. ^a^*P*-value < 0.05, ^b^*P*-value < 0.01. BMI, body mass index; DAS28, Disease Activity Score in 28 joints; HRT, hormone replacement therapy; HTA, hypertensive agent; Log, logarithmically transformed; L, logarithmically transformed; OHA, oral hypoglycemic agent; py, pack year.

### Relationships of obesity measures with carotid artery atherosclerosis in African black and Caucasian women with RA

Due to consistent differences in the relationships of obesity measures with cIMT and carotid artery plaque between African black and Caucasian women with RA (see below), the respective measures were not associated with atherosclerosis upon analysis of the data in all patients or African black and Caucasian women combined.

**Figure 1:**
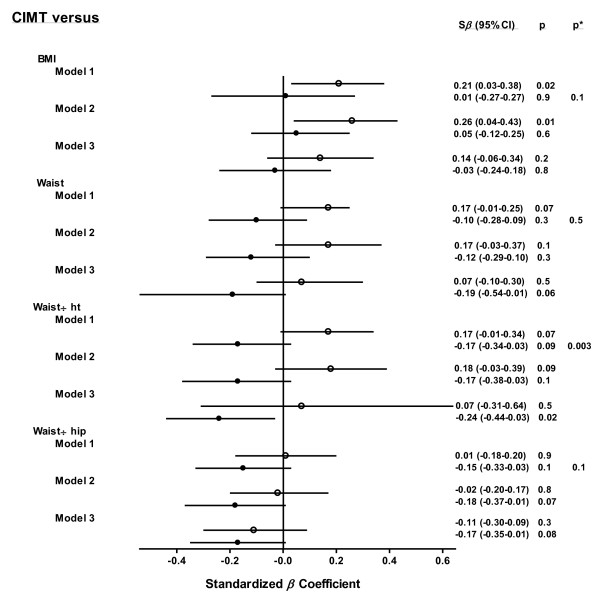
**Obesity measure - carotid intima-media thickness relations in African women with RA**. Disparities in the relationships of obesity measures with ultrasonographically determined carotid artery intima-media thickness between black and Caucasian women with RA after adjustment for age and healthcare (model 1), age, healthcare and non-metabolic risk factors including lifestyle factors, current disease activity (DAS28), cumulative disease activity (deformed joints), prednisone use, hypothyroidism, hormone replacement therapy and cardiovascular drug use (model 2) and age, healthcare center and metabolic risk factors comprising systolic and diastolic blood pressure and HDL cholesterol, triglyceride and glucose concentrations (model 3). Open and closed circles and their horizontal crossing lines represent the odds ratios and 95% confidence intervals for the relationships in Caucasian and black women, respectively. BMI, body mass index; CI, confidence interval; cIMT, carotid intima-media thickness; DAS28, Disease Activity Score in 28 joints; HDL, high density liporotein; ht, height; RA, rheumatoid arthritis; S*β*, standardized regression coefficient.

Results of age and health care adjusted stratified analyses for cIMT are shown in Figure 1. The association of waist-to-height ratio with cIMT differed in African black compared to Caucasian women (*P *for interaction < 0.003). BMI was significantly related to cIMT in African Caucasian women. By contrast, none of the obesity measures were associated with cIMT among African black women.

**Figure 2:**
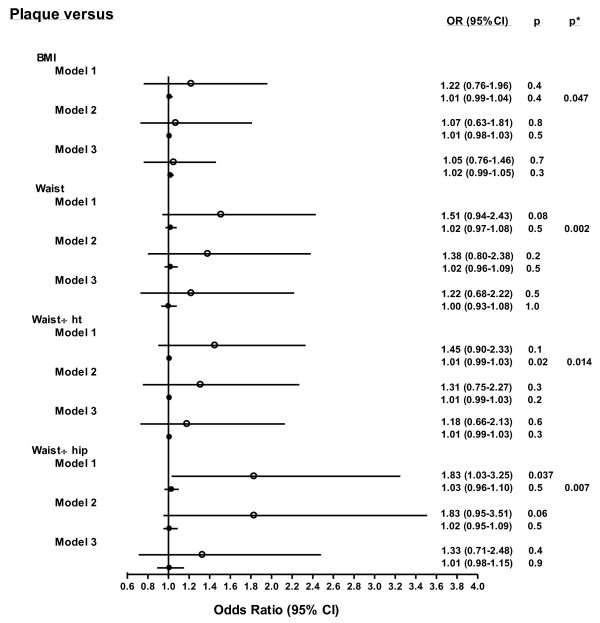
**Obesity measure - carotid plaque relations in African women with RA**. Disparities in the relationships of obesity measures with ultrasonographically determined carotid artery plaque between black and Caucasian women with RA after adjustment for age and healthcare (model 1), age, healthcare and non-metabolic risk factors including lifestyle factors, current disease activity (DAS28), cumulative disease activity (deformed joints), prednisone use, hypothyroidism, hormone replacement therapy and cardiovascular drug use (model 2) and age, healthcare center and metabolic risk factors comprising systolic and diastolic blood pressure and HDL cholesterol, triglyceride and glucose concentrations (model 3). Open and closed circles and their horizontal crossing lines represent the odds ratios and 95% confidence intervals for the relationships in Caucasian and black women, respectively. BMI, body mass index; CI, confidence interval; DAS28, Disease Activity Score in 28 joints; HDL, high density liporotein; ht, height; OR, odds ratio; RA, rheumatoid arthritis.

Results of age and health care adjusted stratified analyses for plaque are shown in Figure 2. The associations of each obesity measure with carotid artery plaque differed in African black compared to Caucasian women (*P *for interaction < 0.05). Waist-to-hip ratio was significantly related to carotid artery plaque in African Caucasian women whereas none of the obesity measures were associated with carotid artery plaque in black women.

### Impact of adjustment for multiple non-metabolic and metabolic risk factors on anthropometric measure - atherosclerosis relations in African Caucasian women with RA

As shown in the Figures, upon adjustment for age, healthcare center and non-metabolic risk factors including lifestyle factors (smoking variable was pack-year history of smoking), current disease activity (DAS28), cumulative disease activity (deformed joints), prednisone use, hypothyroidism, hormone replacement therapy and use of cardiovascular drugs, the associations of BMI with cIMT (Figure 1) and of waist-to-hip ratio with carotid plaque (Figure 2) were not materially altered. By contrast, upon adjusting for sociodemographic characteristics and metabolic risk factors comprising systolic and diastolic blood pressure and HDL cholesterol, triglyceride and glucose concentrations, the associations of BMI with cIMT and waist-to-hip ratio with plaque were markedly attenuated. Further, in African black women, a high waist-to-height ratio was significantly associated with a small cIMT (Figure 1).

## Discussion

In this study, African black women with RA that form part of a developing population sustained a markedly increased adiposity burden compared to their Caucasian counterparts. The associations of obesity measures with metabolic risk factors were as strong in African black compared to Caucasian women with RA. By contrast, obesity measures were not related to carotid atherosclerosis in African black women with RA whereas BMI and the waist-to-hip ratio were associated with atherosclerosis among Caucasian women who belong to a developed population and these relationships were explained by metabolic risk factors.

Relationships between adiposity measures and cardiovascular risk and disease could be expected to be stronger in patients with RA compared to non-RA subjects. In non-RA subjects, a high BMI can reflect an increased body fat mass or a large muscle mass, factors that are each associated with opposite outcomes in cardiovascular disease [19]. However, Stavropoulos-Kalinoglou and colleagues recently reported that patients with RA experience a 4.3% increase in body fat mass for a given BMI compared to healthy individuals [34]. This is most probably attributable to the presence of rheumatoid cachexia that affects nearly two thirds of patients with RA [15-17,34].

The most striking finding in our analysis was revealed upon analyzing the associations of obesity measures with carotid artery plaque among African black women with RA. For each of the adiposity measures, the OR for plaque per 1 SD increase in obesity measure was between 1.00 and 1.03 and the 5 to 95% confidence intervals were very small (see Figure 2). This evident consistent lack of relationships between obesity measures and atherosclerosis could not be attributed to reduced metabolic cardiovascular risk in African black women with RA. Indeed, BMI and waist-to-height ratio were higher in African black compared to Caucasian women and the associations of obesity measures with metabolic risk factors were similar in both groups. Ultrasonographically determined carotid artery plaque is associated with a 10-year cardiovascular event rate risk of 39% in non-RA subjects [35] and reportedly predicts incident ACVD in patients with RA irrespective of population origin [36,37]. Our findings indicate that excess adiposity in patients with RA from developing groups of black African descent does not as yet translate in severe atherosclerosis as reflected by the presence of plaque (see below). Alternatively or additionally, genetic differences between the African black and Caucasian populations may underlie our findings. Indeed, in studies that included African black and European subjects, a differential ethnic distribution of genetic polymorphisms that are associated with obesity [38] as well as those related to cardiovascular disease [39] were found. Either way, our results are congruous with the distinctly low ACVD event rates despite highly prevalent obesity as reported in non-RA African black women [25-30]. Further, the data analysis in the present study indicates that the presence of RA does not alter this current absence of adiposity related atheroma.

Our observation that obesity measures were also not associated with increased cIMT among African black women with RA further substantiates the absence of excess adiposity-related atherogenesis in this patient population. In addition, the waist-to-height and waist-to-hip ratios tended (*P *= 0.09 and 0.1, respectively) to be negatively associated with cIMT and the waist-to-height ratio was significantly (*P *= 0.02) and negatively associated with cIMT upon adjustment for metabolic risk factors. This finding is reminiscent of the reported paradoxical inverse relationships between BMI and ACVD mortality as reported in patients with RA from developed populations [15]. Important potential confounders in this context are smoking and the use of cardiovascular drugs [18]. The latter variables did not alter the relationships of waist-to-height and waist-to-hip ratios with cIMT in the present investigation.

The burden of atherosclerosis was as extensive in African black compared to Caucasian women with RA in this study. These results indicate that systematic cardiovascular risk assessment should be performed irrespective of adiposity extent in African black women with RA.

Published reports on the associations between obesity measures and atherosclerosis in patients with RA, even in those that form part of developed populations, are limited. In African Caucasian women with RA, we found relationships of obesity measures with cIMT and internal carotid artery and carotid artery bulb plaque. cIMT and plaque constitute different phenotypes of atherosclerosis and are biologically and genetically distinct [40-45]. The cIMT constitutes approximately 80% media and approximately 20% intima [41]. Intima-media thickening results mostly from adaptive responses of medial cells to blood pressure and age [41] and is associated with stroke risk factors and stroke prevalence [43-45]. On the other hand, carotid artery plaque occurs as a consequence of intimal pathology [40] and reflects an advanced stage of atherosclerosis that is more closely related to coronary artery disease risk factors and coronary heart disease prevalence [41,43]. Importantly also in the present context, the INTERHEART study investigators found that waist-to-hip ratio was more strongly associated with myocardial infarction than BMI [21] whereas in another recent large study performed in Finland and reported by Hu and colleagues, BMI but not waist-to-hip ratio enhanced the risk for stroke among female participants [46]. Our results in African Caucasian women with established RA are in keeping with these reported findings in that BMI was associated with blood pressure and cIMT whereas waist-to-hip ratio was related to lipids and plaque. In addition, metabolic cardiovascular risk factors explained the relationships of adiposity measures with carotid atherosclerosis thereby supporting the presence of an obesity effect. A differential effect among the individual metabolic risk factors on the associations of BMI with cIMT and waist-to-hip ratio with plaque could not be assessed as lipid characteristics and blood pressure variables were collinear (data not shown). Taken together, our results indicate that in patients with established RA from a developed population, BMI and waist-to-hip ratio are associated with different metabolic risk factor profiles, reflect different aspects of carotid artery atherosclerosis and, therefore, both can be helpful in ACVD risk assessment.

Our study has further limitations. Our patients were exclusively women and the cross-sectional design of our investigation precludes drawing inferences on the direction of causality. Further, characteristics that can be important in the present context and were not recorded include menopausal status and cumulative prednisone dose. Also, clinical measures of abdominal obesity do not distinguish between visceral and subcutaneous fat. Imaging studies (for example, computerized tomography) will be needed to determine the relative impact of visceral as compared to deep and superficial subcutaneous fat at the abdominal level on atherogenesis in patients with RA [47,48]. Visceral fat is particularly strongly associated with ACVD risk [20-22]. Circulating triglyceride concentrations reflect visceral fat mass [29] and these did not differ in African black compared to Caucasian women with RA in the current investigation. Finally, as applies in previously reported investigations on cardiovascular risk in non-RA as well as RA subjects, many relationships were evaluated. However, our main findings each originate in confounder adjusted multivariable regression models.

## Conclusions

Although women with established RA from developing groups of African descent experience a larger obesity burden compared to their Caucasian counterparts, this is currently not as yet associated with enhanced carotid atherosclerosis. Additionally and contrastingly, we report for the first time evidence that supports the use of clinical obesity measures including BMI and waist-to-hip ratio in the assessment of ACVD and risk among Caucasian women with established RA from a developed population.

## Abbreviations

ACVD: atherosclerotic cardiovascular disease; β: regression coefficient; BMI: body mass index; CI: confidence interval; cIMT: carotid intima-media thickness; CRP: C-reactive protein; DAS 28: disease activity in 28 joints; DMARD: disease modifying drugs for rheumatic disease; ESR: erythrocyte sedimentation rate; HDL: high density lipoprotein; LDL: low density lipoprotein; PG: population grouping; OR: odds ratio; RA: rheumatoid arthritis; S and Std: standardized; SD: standard deviation.

## Competing interests

The authors declare that they have no competing interests.

## Authors' contributions

AS contributed to the conception and design, data acquisition, interpretation of the data and revising the manuscript and performed the carotid ultrasound examinations in public healthcare patients. GRN and AJW contributed to the conception and design and analysis and interpretation of the data. PHD contributed to the conception and design and data acquisition, performed the statistical analysis and drafted the manuscript. All authors read and approved the final manuscript.

## References

[B1] Meune C Touze E Trinquart L Allanore Y High risk of clinical cardiovascular events in rheumatoid arthritis: levels of associations of myocardial infarction and stroke through a systematic review and meta-analysis Arch Cardiovasc Dis 2010 103 253 261 20656636 10.1016/j.acvd.2010.03.007

[B2] Avina-Zubieta Antonio J Choi HK Sadatsafavi M Etminan M Esdaile JM Lacaille D Risk of cardiovascular mortality in patients with rheumatoid arthritis: a meta-analysis of observational studies Arthritis Rheum 2008 59 1690 1697 19035419 10.1002/art.24092

[B3] Dessein PH Joffe BI Veller MG Stevens BA Tobias M Reddi K Stanwix AE Traditional and nontraditional cardiovascular risk factors are associated with atherosclerosis in rheumatoid arthritis J Rheumatol 2005 32 435 442 15742434

[B4] Gonzalez-Gay MA Gonzalez-Juanatey C Pineiro A Garcia-Porrua C Testa A Llorca J High-grade C-reactive protein elevation correlates with accelerated atherogenesis in patients with rheumatoid arthritis J Rheumatol 2005 32 1219 1223 15996055

[B5] del Rincon I Freeman GL Haas RW O'Leary DH Escalante A Relative contribution of cardiovascular risk factors and rheumatoid arthritis clinical manifestations to atherosclerosis Arthritis Rheum 2005 52 3413 3423 16255018 10.1002/art.21397

[B6] Chung CP Oeser A Raggi P Gebretsadik T Shintani AK Sokka T Pincus T Avalos I Stein CM Increased coronary-artery atherosclerosis in rheumatoid arthritis: relationship to disease duration and cardiovascular risk factors Arthritis Rheum 2005 52 3045 3053 16200609 10.1002/art.21288

[B7] Roman MJ Moeller E Davis A Paget SA Crow MK Lockshin MD Sammaritano L Devereux RB Schwartz JE Levine DM Salmon JE Preclinical carotid atherosclerosis in patients with rheumatoid arthritis Ann Intern Med 2006 144 249 256 16490910 10.7326/0003-4819-144-4-200602210-00006

[B8] Kremers HM Crowson CS Themeau TM Roger VL Gabriel SE High ten-year risk of cardiovascular disease in newly diagnosed rheumatoid arthritis patients: a population-based cohort study Arthritis Rheum 2008 58 2268 2274 18668561 10.1002/art.23650 PMC2929699

[B9] Wolfe F Michaud K The risk of myocardial infarction and pharmacologic and nonpharmacologic myocardial infarction predictors in rheumatoid arthritis: a cohort and nested case-control analysis Arthritis Rheum 2008 58 2612 2621 18759273 10.1002/art.23811

[B10] Solomon DH Kremer J Curtis JR Hochberg MC Reed G Tsao P Farkouh ME Setoquchi S Greenberg JD Explaining the cardiovascular risk associated with rheumatoid arthritis: traditional risk factors versus markers of rheumatoid arthritis severity Ann Rheum Dis 2010 69 1920 1925 20444756 10.1136/ard.2009.122226 PMC2963658

[B11] Gonzalez-Gay MA Gonzalez-Juanatey C Lopez-Diaz MJ Pineiro A Garcia-Porrua C Miranda-Filloy JA Ollier WE Martin J Llorca J HLA-DRB1 and persistent chronic inflammation contribute to cardiovascular events and cardiovascular mortality in patients with rheumatoid arthritis Arthritis Rheum 2007 57 125 132 17266100 10.1002/art.22482

[B12] Toms TE Panoulas VF Smith JP Douglas KM Metsios GS Stavropoulos-Kalinoglou A Kitas GD Rheumatoid arthritis susceptibility genes associate with lipid levels in patients with rheumatoid arthritis Ann Rheum Dis 2011 70 1025 1032 21398331 10.1136/ard.2010.144634

[B13] Stavropoulos-Kalinoglou A Metsios GS Panoulas VF Douglas KM Nevill AM Jamurtas AZ Kita M Koutedakis Y Kitas GD Associations of obesity with modifiable risk factors for the development of cardiovascular disease in patients with rheumatoid arthritis Ann Rheum Dis 2009 68 242 245 18677010 10.1136/ard.2008.095596

[B14] Dessein PH Joffe BI Insulin resistance and impaired beta cell function in rheumatoid arthritis Arthritis Rheum 2006 54 2765 2775 16947779 10.1002/art.22053

[B15] Maradit Kremers H Nicola PJ Crowson CS Ballman KV Gabriel SE Prognostic importance of low body mass index in relation to cardiovascular mortality in rheumatoid arthritis Arthritis Rheum 2004 50 3450 3457 15529378 10.1002/art.20612

[B16] Escalante A Haas RW del Rincon I Paradoxical effect of body mass index on survival in rheumatoid arthritis Arch Intern Med 2005 165 1624 1629 16043681 10.1001/archinte.165.14.1624

[B17] Kitas GD Gabriel SE Cardiovascular disease in rheumatoid arthritis: state of the art and future perspectives Ann Rheum Dis 2011 70 8 14 21109513 10.1136/ard.2010.142133

[B18] Stavropoulos-Kalinoglou A Metsios GS Koutedakis Y Kitas GD Obesity in rheumatoid arthritis Rheumatology 2011 50 450 462 20959355 10.1093/rheumatology/keq266

[B19] Romero-Corral A Somers VK Sierra-Johnson J Jensen MD Thomas RJ Squires RW Allison TG Korinek J Lopez-Jimenez F Diagnostic performance of body mass index to detect obesity in patients with coronary artery disease Eur Heart J 2007 28 2087 2093 17626030 10.1093/eurheartj/ehm243

[B20] Lee CMY Huxley RR Wildman RP Woodward M Indices of abdominal obesity are better discriminators of cardiovascular risk factors than BMI: a meta-analysis J Clin Epidemiol 2008 61 646 653 18359190 10.1016/j.jclinepi.2007.08.012

[B21] Yusuf S Hawken S Ounpuu S Bautista L Franzosi G Commerford P Lang CC Rumboldt Z Onen CL Lisheng L Tanomsup S Wangai P Jr Razak F Sharma AM Anand SS INTERHEART Study Investigators Obesity and the risk of myocardial infarction in 27 000 participants from 52 countries: a case-control study Lancet 2005 366 1640 1649 16271645 10.1016/S0140-6736(05)67663-5

[B22] Browning LM Hsieh SD Ashwell M A systematic review of waist-to-height ratio as a screening tool for the prediction of cardiovascular disease and diabetes: 0.5 could be a suitable global boundary value Nutr Res Rev 2010 23 247 269 20819243 10.1017/S0954422410000144

[B23] Yusuf S Hawken S Ounpuu S Dans T Avezum A Lanas F McQueen M Budaj A Pais P Varigos J Lisheng L INTERHEART Study Investigators Effect of potentially modifiable risk factors associated with myocardial infarction in 52 countries (the INTERHEART study): case-control study Lancet 2004 364 937 952 15364185 10.1016/S0140-6736(04)17018-9

[B24] Solomon A Christian BF Norton GR Woodiwiss AJ Dessein PH Risk factor profiles for atherosclerotic cardiovascular disease in black and other Africans with established rheumatoid arthritis J Rheumatol 2010 37 953 960 20231201 10.3899/jrheum.091032

[B25] Steyn K Sliwa K Hawken S Commerford P Onen C Damasceno A Ounpuus A Yusuf S INTERHEART investigators in Africa Risk factors associated with myocardial infarction in Africa. The INTERHEART Africa Study Circulation 2005 112 3554 3561 16330696 10.1161/CIRCULATIONAHA.105.563452

[B26] Mayosi BM Flisher AJ Lalloo UG Sitas F Tollman SM Bradshaw D The burden of non-communicable diseases in South Africa Lancet 2009 374 934 947 19709736 10.1016/S0140-6736(09)61087-4

[B27] Solomon A Christian BF Woodiwiss AJ Norton GR Dessein PH Burden of depressive symptoms in South African public healthcare patients with established rheumatoid arthritis: a case-control study Clin Exp Rheumatol 2011 29 506 512 21640040

[B28] Puoane T Steyn K Bradshaw D Laubscher R Fourie J Lambert V Mbananga N Obesity in South Africa: The South African demographic and health survey Obes Res 2002 10 1038 1048 12376585 10.1038/oby.2002.141

[B29] Goedecke JH Jennings CL Lambert EV Obesity in South Africa Chronic Diseases of Lifestyle in South Africa since 2005 http://www.mrc.ac.za/chronic/cdlchapter7.pdf

[B30] Walker ARP Sareli P Coronary heart disease: outlook for Africa J R Soc Med 1997 90 23 27 9059377 10.1177/014107689709000108 PMC1296111

[B31] Arnett FC Edworthy SM Bloch DA McShane DJ Fries JF Cooper NS Healey LA Kaplan SR Liang MH Luthra HS The American Rheumatism Association 1987 revised criteria for the classification of rheumatoid arthritis Arthritis Rheum 1988 31 315 324 3358796 10.1002/art.1780310302

[B32] Gepner AD Korcarz CE Aeschlimann SE LeCaire TJ Palta M Tzou WS Stein JH Validation of a carotid intima-media thickness border detection program for use in an office setting J Am Soc Echocardiogr 2006 19 223 228 16455429 10.1016/j.echo.2005.09.006

[B33] Touboul PJ Hennerici MG Meairs S Adams H Amarenco P Bornstein N Csiba L Desvarieux M Ebrahim S Fatar M Hernandez-Hernandez R Jafi M Kownator S Prati P Rundek T Sitzer M Schminke U Tardif JC Taylor A Vicaut E Woo KS Zannad F Zureik M Mannheim carotid intima-media thickness consensus (2004-2006). An update on behalf of the Advisory Board of the 3^rd ^and 4^th ^Watching the Risk Symposium, 13^th ^and 15^th ^European Stroke Conferences, Mannheim, Germany, 2004, and Brussels, Belgium, 2006 Cerebrovasc Dis 2007 23 75 80 17108679 10.1159/000097034

[B34] Stavropoulos-Kalinoglou A Metsios GS Koutedakis Y Nevill AM Douglas KM Jamurtas A Veldhuijzen van Zanten JJCS Labib M Kitas GD Redefining overweight and obesity in rheumatoid arthritis patients Ann Rheum Dis 2007 66 1316 1321 17289757 10.1136/ard.2006.060319 PMC1994320

[B35] Belcaro G Nicolaides AN Ramaswami G Cesarone MR De Sanctis M Incandela L Ferrari P Geroulakos G Barsotti A Griffin M Dhanjil S Sabetai M Bucci M Martines G Carotid and femoral ultrasound morphology screening and cardiovascular events in low risk subjects: a 10-year follow-up study (the CAFES-CAVES study (1)) Atherosclerosis 2001 156 379 387 11395035 10.1016/s0021-9150(00)00665-1

[B36] Gonzalez-Juanatey C Llorca J Martin J Gonzalez-Gay MA Carotid intima-media thickness predicts the development of cardiovascular events in patients with rheumatoid arthritis Semin Arthritis Rheum 2009 38 366 371 18336869 10.1016/j.semarthrit.2008.01.012

[B37] Evans MR Escalante A Battafarano DF Freeman GL O'Leary DH del Rincon I Carotid atherosclerosis predicts incident acute coronary syndrome in rheumatoid arthritis Arthritis Rheum 2011 63 1211 1220 21305526 10.1002/art.30265 PMC3286362

[B38] Siffert W Forster P Jockel KH Mvere DA Brinkmann B Naber C Crookes R Du P Heyns A Epplen JT Fridey J Freedman BI Muller N Stolke D Sharma AM Al Moutaery K Grosse-Wilde H Beurman B Ehrlich T Ahmad HR Horsthempke B Du Toit ED Tiilikainen A Ge J Wang Y Rosskopf D Worldwide ethnic distribution of the G protein beta3 subunit 825T allele and its association with obesity in Caucasian, Chinese, and Black African individuals J Am Soc Nephrol 1999 10 1921 30 10477144 10.1681/ASN.V1091921

[B39] Predazzi IM Martinez-Labarga C Vecchione L Mango R Ciccacci C Amati F Ottoni C Crawford MH Rickards O Romeo F Novelli G Population differences in allele frequencies at the OLR1 locus may suggest geographic disparities in cardiovascular risk events Ann Hum Biol 2010 37 136 48 19961348 10.3109/03014460903393857

[B40] Simon A Megnien J-L Chironi G The value of carotid intima-media thickness for predicting cardiovascular risk Arterioscler Thromb Biol 2010 30 182 185 10.1161/ATVBAHA.109.196980 19948842

[B41] Riccio SA House AA Spence JD Fenster A Parraga G Carotid ultrasound phenotypes in vulnerable populations Cardiovasc Ultrasound 2006 4 44 17101043 10.1186/1476-7120-4-44 PMC1657034

[B42] Johnsen SH Mathiesen EB Joakimsen O Stensland E Wilsgaard T Lochen M-L Njolstad I Arnesen E Carotid atherosclerosis is a stronger predictor of myocardial infarction in women than in men: a 6-year follow-up study of 6226 persons: The Tromso Study Stroke 2007 38 2873 2880 17901390 10.1161/STROKEAHA.107.487264

[B43] Ebrahim S Papacosta O Whincup P Wannamethee G Walker M Nicolaides AN Dhanjil S Griffin M Belcaro G Rumley A Lowe GDO Carotid plaque, intima media thickness, cardiovascular risk factors, and prevalent cardiovascular disease in men and women: The British Regional Heart Study Stroke 1999 30 841 850 10187889 10.1161/01.str.30.4.841

[B44] Spence JD Hegele RA Noninvasive phenotypes of atherosclerosis Arterioscler Thromb Vasc Biol 2004 24 e188 15528487 10.1161/01.ATV.0000146160.22637.33

[B45] Johnsen SH Mathiesen EB Carotid plaque compared with intima-media thickness as a predictor of coronary and cerebrovascular disease Curr Cardiol Rep 2009 11 21 27 19091171 10.1007/s11886-009-0004-1

[B46] Hu G Tuomilehto J Silventoinen K Sartri C Mannisto S Jousilahti P Body mass index, waist circumference, and waist-hip ratio on the risk of total and type-specific stroke Arch Intern Med 2007 167 1420 1427 17620537 10.1001/archinte.167.13.1420

[B47] Giles JT Allison M Blumenthal RS Post W Gelber AC Petri M Tracy R Szklo M Bathon JM Abdominal adiposity in rheumatoid arthritis: association with cardiometabolic risk factors and disease characteristics Arthritis Rheum 2010 62 3173 3182 20589684 10.1002/art.27629 PMC2962724

[B48] Goedecke JH Levitt NS Lambert EV Utzschneider KM Faulenbach MV Dave JA West S Victor H Evans J Olsson T Walker BR Seckl J Kahn SE Differential effects of abdominal adipose tissue distribution on insulin sensitivity in black and white South African women Obesity 2009 17 1506 1512 19300428 10.1038/oby.2009.73

